# The epidemiology of shoulder dislocations in Oslo

**DOI:** 10.1111/j.1600-0838.2011.01300.x

**Published:** 2011-12

**Authors:** S Liavaag, S Svenningsen, O Reikerås, M Enger, T Fjalestad, A H Pripp, J I Brox

**Affiliations:** 1 Department of Orthopedic Surgery, Sørlandet HospitalArendal, Norway; 2 Department of Orthopedic Surgery, Oslo University HospitalRikshospitalet, Norway; 3 Oslo skadelegevakt, Oslo University HospitalUllevaal, Norway; 4 Department of Orthopedic Surgery, Oslo University HospitalAker, Norway; 5 Biostatistics and Epidemiology Unit, Oslo University HospitalRikshospitalet, Norway

**Keywords:** shoulder dislocations, general population, Norway, Oslo

## Abstract

There are few previous studies on the incidence of shoulder dislocation in the general population. The aim of the study was to report the incidence of acute shoulder dislocations in the capital of Norway (Oslo) in 2009. Patients of all ages living in Oslo, sustaining a dislocation of the glenohumeral joint, were identified using electronic diagnosis registers, patient protocols, radiological registers of the hospitals, and the Norwegian Patient Register (NPR). The overall incidence rate was 56.3 [95% confidence interval (CI) 50.2–62.4] per 100 000 person-years, with rates of 82.2 (95% CI 71.7–92.8) and 30.9 (95% CI 24.5–37.3) in men and women, respectively. The incidence of primary dislocations was 26.2 (95% CI 22.1–30.4). The overall incidence of shoulder dislocations in Oslo was higher than previously reported incidences. The incidence of primary dislocations was also higher than that in previously reported studies for the general population but it was close to the incidence reported in Malmø, Sweden.

The few previous studies on the incidence of shoulder dislocation in the general population report incidences ranging from 11.2 to 23.9 per 100 000 person-years (Simonet et al., 1984; Kroner et al., 1989; Nordqvist & Petersson, 1995; Zacchilli & Owens, 2010).

Incidences of shoulder dislocation in Scandinavian populations have been studied in Sweden and Denmark but not in Norway (Kroner et al., 1989; Nordqvist & Petersson, 1995). The prevalence of shoulder dislocation in the Swedish population was found to be at least 1.7% (Hovelius, 1982). In Aarhus, Denmark, the incidences of all dislocations and primary dislocations were reported to be 17 and 12.3 per 100 000 person-years, respectively (Kroner et al., 1989). In Malmø, Sweden, the incidence of primary dislocations was reported to be 23.9 per 100 000 person-years, which is equal to the estimated overall incidence rate of shoulder dislocation in the United States reported in 2010 (Nordqvist & Petersson, 1995; Zacchilli & Owens, 2010). The aim of the present study was to investigate the incidence of acute shoulder dislocation in Oslo and to compare it with findings in previous studies in Scandinavia and the USA. We also wanted to compare estimated incidences from our manually controlled sample with estimates based on data available from a public medical electronic database to see whether the results are consistent or not.

## Patients and Methods

**Fig. 1:**
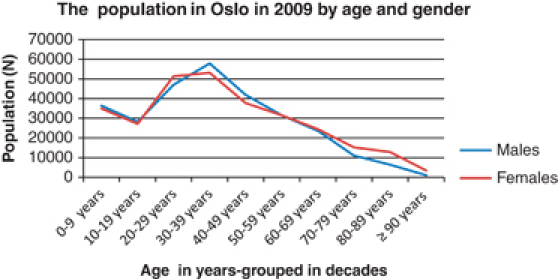
Age and gender distribution of the Oslo populations in 2009.

Oslo, the capital of Norway, had a population of 575 475 inhabitants on January 1, 2009 (Statistics Norway, 2009). Figure 1 shows the age and gender distribution in the Oslo population in 2009. Patients with an acute shoulder dislocation are admitted and treated in one of three public hospitals in the city (two of these hospitals were merged in 2009). Patients are usually admitted first to the emergency ward or possibly to an outpatient ward integrated in the hospital, and most patients are treated at this level of care. Some patients need hospitalization. The largest outpatient clinic in Oslo is an integrated unit of the orthopedic department of Oslo University Hospital, Ullevaal, but serves patients from all hospital areas of Oslo. Inhabitants of the outskirts of the city may be treated at a clinic in a neighboring municipality. This clinic was also asked to report their data for patients with the diagnosis of acute shoulder dislocation, but with an address in Oslo. Three private clinics in Oslo treat surgery emergency cases, and they were also asked to report their acute shoulder dislocations, but only one clinic reported cases in 2009. In all the hospitals and clinics, an electronic diagnosis was used to identify patients with the ICD-10 code S43.0: acute shoulder dislocation. If the patients were transferred from the emergency ward or outpatient clinic to hospitalization in the surgical or the orthopedic department in the same or another hospital, they were only registered once (at the initial place of admission). In addition, the diagnosis was checked against the x-ray and medical reports. Gender, age in years, primary or recurrent dislocation, radiographic documentation, reduction before or after arrival in the clinic and referral to another department or hospital (with the name of the clinic) were all recorded. Physicians in the clinics were responsible for identifying the diagnosis in their diagnosis register, x-ray register and medical records. In one of the clinics a nurse was responsible. A few patients had multiple dislocations within the same year. It was not always clear whether these were new dislocations or a follow-up and we decided only to record the first occurrence within the same calendar year for estimating the incidence of shoulder dislocations. All the data were received without personal identification.

The Norwegian Patient Register (NPR) is a database maintained by the Norwegian Directorate of Health. It contains activity data for all the public and private hospitals in Norway (Norsk pasientregister, 2009). Reporting is mandatory. Medical data are coded according to the International Classification of Diseases system, 10th Revision (ICD-10), but NPR does not receive information about whether the dislocations are primary or recurrent.

The register uses a cryptic identifier for every patient, thus making it possible to exclude any multiple registrations of a patient who is transferred between hospitals. We queried the NPR by age and sex using ICD-10 code S43.0 to determine the number of patients with acute shoulder dislocation in 2009. The age categories used were: <20 years, decades up to 80, and 80 years or older. Events were limited to the “first annual occurrence” of acute shoulder dislocation for each patient to exclude multiple coding of the same injuries. Patients with address outside Oslo were excluded. We used the population of Oslo on January 1 (Statistics Norway, 2009) as the population at risk. All the data were received without personal identification.

The study was performed in accordance with the ethical standards of the revised Helsinki declaration of 2000, and reported to our institution's scientific board.

### Statistics

Descriptive statistics are number of cases and percentages, or median and range, unless otherwise stated. Differences between groups were analyzed using the Mann–Whitney or chi-square test as appropriate. Annual incidence rates per 100 000 person-years were estimated with a 95% confidence interval using the normal approximation to the Poisson distribution.

## Results

Limited to the first annual occurrence for each patient, 324 patients living in Oslo were recorded with an acute shoulder dislocation in 2009; 279 of the patients (86.1%) were treated at the largest outpatient clinic (OLV). Two thirds of the dislocations occurred in male (72.2%, n=234) and one-third in female patients (27.8%, n=90). Confirmed primary dislocations were reported in 151 patients (46.6%) and recurrence in 134 patients (41.4%), while in the remaining 39 patients (12%), the type of dislocation could not be confirmed. If we include only the 285 patients with reported direction of the dislocation, the percentage of primary dislocations would be 53%.

In 216 patients (66.7%) reductions were documented to have been performed in the emergency department, in 79 of 324 dislocations (24.4%) reductions were performed before arrival at the emergency department, 2 (0.6%) were reported with spontaneous reduction while waiting for treatment in the clinic, and for 27(8.3%) explicit information on reduction is missing.

The dislocation was verified by x-ray imaging before reduction in 216 of the dislocations (66.7%), and for 18 patients (5.6%), information on x-ray verification before reduction is missing.

In 18 patients, the age group was only recorded in decades. The age in years for these 18 patients was set equal to the mean age for their given age group. The median age in years was 34 (range 16–95 years) for all patients, 54 (range 16–95) for women, and 30 (range 16–89) years for men (*P*<0.001). The median age for women with confirmed primary dislocation was 66 (range 19–95), and for women with recurrence, it was 30 (range 16–83) years (*P*<0.001). For men, the median age was 37 (range 16–89) years for those with primary dislocation and 27 (range 17–66) for those with recurrence (*P*<0.001).

The age-adjusted dislocation rates and the distributions of primary and recurrent dislocations in different age groups are given in Table 1. We found an overall incidence rate of 56.3 (95% CI 50.2–62.4) per 100 000 person-years. The incidence rates for men and women were 82.2 (95% CI 71.7–92.8) and 30.9 (95% CI 24.5–37.3) per 100 000 person-years, respectively. The incidence of confirmed primary dislocations was 26.2 (95% CI 22.1–30.4), with 34.8 (95% CI 27.9−41.7) and 17.9 (95% CI 13.0−22.7) per 100 000 person-years for men and women, respectively.

**Table 1:** Number of dislocations and annual incidence of shoulder dislocations in Oslo in 2009 (NPR data and incidence rates based on the NPR data are in brackets)

Sex	Age group (years)	Population January 01, 2009	Number of dislocations in total	Annual incidence per 100 000	Number of primary dislocations	Annual incidence “Primary” per 100 000
	0–19	64 481	17 (11)	26.4 (17.1)	6	9.3
	20–29	46 994	96 (108)	204.3 (229.8)	31	66.0
	30–39	57 894	48 (54)	82.9 (93.3)	18	31.1
Men	40–49	41 911	26 (30)	62.0 (71.6)	11	26.3
	50–59	31 474	15 (25)	47.7 (79.4)	9	28.6
	60–69	23 469	16 (12)	68.2 (51.1)	9	38.4
	70–79	10 963	7 (8)	63.9 (73.0)	6	54.7
	≥80	7321	9(9)	122.9 (122.9)	9	122.9
	0–19	61 939	5 (6)	8.1 (9.7)	1	1.6
	20–29	51 235	14 (17)	27.3 (33.2)	5	9.8
	30–39	53 059	16 (15)	30.2 (28.3)	8	15.1
Women	40–49	37 713	6 (6)	15.9 (15.9)	1	2.7
	50–59	31 419	10 (11)	31.8 (35.0)	6	19.1
	60–69	24 378	8 (12)	32.8 (49.2)	7	28.7
	70–79	15 132	13 (15)	85.9 (99.1)	11	72.7
	≥80	16 093	18 (21)	111.9 (130.5)	13	80.8
In total		575 475	324 (360)	56.3 (62.6)	151	26.2

NPR, Norwegian Patient Register.

**Fig. 2:**
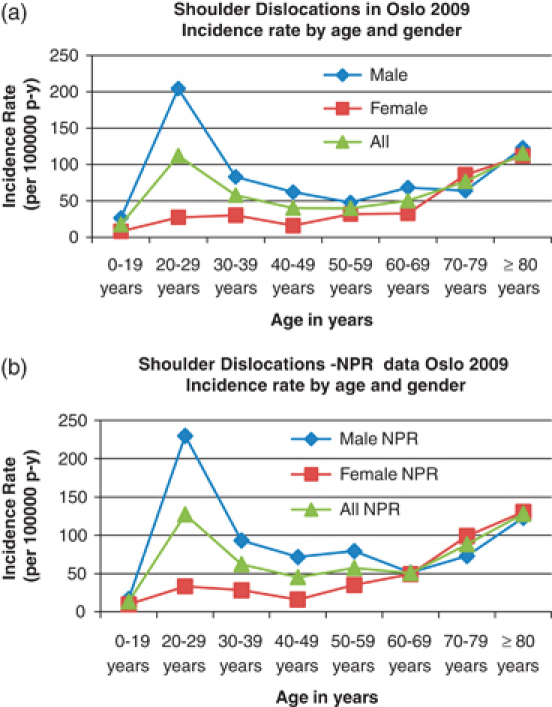
Incidence rates of all shoulder dislocations (per 100 000 person-years) by age and gender in Olso.

The highest incidence of dislocation in male patients was during the third decade of life (20–29 years old). Although the female patients had a small peak late in the second decade of life, the major trend was a gradually increasing frequency with increasing age. We found a gradual increase in the incidence of dislocations in both genders after they had passed the age of 50 (Fig. 2a). Approximately one-third (29.6%, *n*=96) of all dislocations occurred in patients older than 49 years, but only 14.5% (*n*=47) occurred in patients above 69 years. We found in total 70 patients with primary dislocations after 50 years of age, of which 33 (47%) were men and 37 (53%) were women. Half of all the dislocations (48.5%, *n*=157) were found in patients below 34 years, and half of the l34 confirmed that recurrences (50.7%, *n*=68) occurred in patients below 28 years of age. Only about 10% (9.7%, *n*=13) of the recurrences were recorded among patients older than 49 years.

### NPR data on acute shoulder dislocations in 2009

Limited to first annual occurrence for each patient, NPR reported 360 patients living in Oslo with an acute shoulder dislocation in 2009, and 291(80.8%) of the patients were treated at OLV. The distribution of the dislocations and the incidence of shoulder dislocation in the different age groups are given in brackets in Table 1. The overall incidence rate of shoulder dislocation based on reported dislocations in NPR was calculated to be 62.6 (95% CI 56.1–69.0) per 100 000 person-years. The incidence rates for men and women were 90.3 (95% CI 79.3–101.4) and 35.4 (95% CI 28.6–42.2) per 100 000 person-years, respectively. Figure 2a and b demonstrate the distribution of incidence rates by age and gender both in our Oslo sample and in the NPR sample, and illustrate the similarity between the two samples.

## Discussion

The overall incidence in the present study was 56.3 (95% CI 50.2–62.4), and the incidence of confirmed primary dislocations was 26.2 (95% CI 22.1–30.4 per 100 000 person-years. The overall incidence is much higher than that reported in previous studies on the general population (Simonet et al., 1984; Kroner et al., 1989; Zacchilli & Owens, 2010). The incidence of primary dislocations is also higher than that in the previous reported studies but close to that reported in Malmø, Sweden (Nordqvist & Petersson, 1995) (Table 2).

**Table 2:** Present study compared with previous studies on population, demography and incidence rates

Study (year published)	Simonet et al. (1984)	Krøner et al. (1989)	Nordquist and Petersson (1995)	Owens et al. (2007)	Owens et al. (2009a, b)	Zacchilli and Owens (2010)	Present study (2010)
Population	USA, Olmsted	Denmark	Sweden	USA	USA	USA	Norway
	Minnesota	Aarhus	Malmø	Military	Military	Electronic	Oslo
	General	General	General	Selected	Selected	Database	General
	Rural/urban	Urban	Urban	Academy	National	National	Urban
Population at risk	880 000	1 268 765	230 056	4134	11 680 893	1.46 billion	575 475
Study period	10 years 1970–1979	5 years 1980–1984	1 year 1987	1 year 2004–2005	9 years 1998–2006	5 years 2002–2006	1 year 2009
Dislocations in total (*n*)						8940	
	124	216	55	18	19 730	349 486 (model)	324
Dislocations primary (*n*, %)			55	12			151
			100%	66.7%	NR	NR	46.7%
Male proportion (%)	66.7%	53.3%	53%	NR	92.5%	71.8%	72.4%
Age(years)	Mean 36.4	Median 51	Mean 44 male 63 female	Mean 20	NR	Mean 35.4	Median 34
Incidence rate per 100 000 person-years							
All dislocations both genders	11.2	17		435	169	23.9	56.3
Primary dislocations both genders	8.2 (*adjusted)	12.3	23.9	290	NR	NR	26.2
All dislocations men	11.2 (*adjusted)	9.1		NR	182	34.9	82.2
Primary dislocations men			27	NR		NR	34.8
All dislocations women	5.0 (*adjusted)	8.0		NR	90	13.3	30.9
Primary dislocations women			22	NR	NR	NR	17.9

* Sex-and age-adjusted to the population structure of the United States in 1980.

NR, not rated.

The distribution of the dislocations according to age and gender seemed very similar in the NPR and in our Oslo sample. The calculated overall incidences agreed fairly well with each other, with incidence rates of 62.6 and 56.3, respectively. We do not know whether the small discrepancy between the NPR sample and our own data is caused by “false-positive dislocations” in the NPR sample or whether we have failed to record “true positive dislocations” in our Oslo sample. It was not possible to check the diagnosis in the NPR data with x-ray registers and medical records in the hospitals because we used no personal identification. However, the results of incidences for shoulder dislocations were close to each other and indicate that the data are valid.

Approximately 80% of the patients were treated at the largest outpatient clinic, which is in accordance with earlier reported results (83%) for forearm fractures in Oslo (Lofthus et al., 2008).

**Fig. 3:**
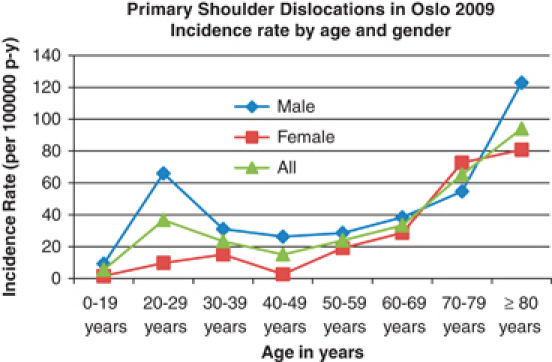
Incidence rates of primary shoulder dislocations (per 100 000 person-years) by age and gender in Oslo.

The strength of the present study is a well-defined population limited to the “first annual occurrence” of acute shoulder dislocation, excluding multiple coding of the same injuries. The incidence rates are reduced to a minimum level. We report incidence rates for primary dislocations and dislocations in total for both genders (Fig. 3). All types of acute dislocation with the need for reduction and/or an x-ray check after reduction were included.

Electronic diagnosis registers in the clinics were used to identify dislocations in our Oslo sample. X-ray imaging before reduction in two-thirds of the patients verified the dislocation, and approximately one-fourths of the patients had already been reduced before arrival. Less than 10% of the patients missed exact information concerning x-ray verification or reduction before arrival. We cannot exclude that a subluxation or another diagnosis might have been mistakenly recorded, but dislocation was verified in most patients by reviewing the medical records and x-ray reports, thus avoiding overestimation of the reported incidence.

With all studies included, there seems to be a tendency toward an increasing incidence rate over the last three decades (Table 2). Like the former Scandinavian studies, we investigated an urban population, but with a higher proportion of young patients with a median age of 34 years (30 in men and 54 in women) compared with a median age of 51 years in the Danish study and a mean age of 44 for males and 63 for females in the Swedish study (Kroner et al., 1989; Nordqvist & Petersson, 1995). We confirmed a higher incidence in males as reported previously in other studies (Simonet & Cofield, 1984; Kroner et al., 1989; Nordqvist & Petersson, 1995; Zacchilli & Owens, 2010). The male proportion of patients (72.4%) was even higher in our study than in the two earlier Scandinavian studies, but was almost identical to the recently published American study (Table 2). The incidence rate in the different age groups showed a bimodal distribution with peaks for young adults and in the elderly for both genders, which is in agreement with previous studies (Simonet et al., 1984; Kroner et al., 1989; Zacchilli & Owens, 2010). The shape of the “distribution curves” (Fig. 2) resembles the shape of curves in a similar figure describing the incidence in the general American population (Zacchilli & Owens, 2010). Kazar and Relovszky (1969) reported that first time dislocations were more common among females after 50 years of age. When we compared the numbers of dislocations for all males and females after 50 years of age, we found that the incidences of primary dislocations were almost equal for both genders.

The organization of the health care system may affect the differences in the incidence reported in the general population between the United States and Oslo, Norway. Norway has a public health care system with access for all citizens, and registrations of diagnoses are mandatory. The National Electronic Injury Surveillance System (NEISS) database used to register and code patients in the US incidence study only included patients at emergency departments (Zacchilli & Owens, 2010). The proportion of patients seeking help in other parts of the health care system (who did not record data to the NEISS database) or those who failed to seek formal medical care remains unknown. It is particularly likely that the last group is larger in the United States, where there is no universal access to health care, and more than 46 million Americans do not have health insurance (Weisz et al., 2008).

Earlier studies have reported very high incidence rates in military and athletic populations, with male sex and young age as two of the main risk factors for injury (Owens et al., 2007; Owens et al., 2009a, b). The incidence of all dislocations reported in the US military was five times higher than the incidence reported for the general population in the United States, and three times higher than the incidence we found (Table 2). Although a high proportion of young physically active males in the military may be a major contributing factor, the differences between the American military and civilian health care systems may also be of importance. The US military population has good access to health care and treatment, and data are collected for all visits (Owens et al., 2009b). Our study confirms that there is a huge difference in the overall incidence of shoulder dislocation between a selected very physically active population and the general population. However, this difference is not as huge as earlier reported especially if we compare with the young and male part of the general population.

In a study from the Israeli Defense Forces Medical Corps between the years of 1978 and 1995, the prevalence rate of subjects with a history of shoulder dislocations in the male proportion between the ages of 22 and 23 years was as high as 42.4 of 10 000 (Milgrom et al., 1998). Hovelius (1978) reported that humeroscapular dislocation, primary or recurrent, was found in 8% of elite ice hockey players in Sweden. Both studies confirm that in a young and physically active population, one must expect a high occurrence of shoulder dislocations if it is possible to carry out a careful and accurate registration of the accidents.

We found a high incidence of dislocations in the elderly population, increasing with increasing age after the sixth decade of life – this agrees with reported incidences of other fall accident injuries (Lofthus et al., 2001; Lofthus et al., 2008).

Several studies have found the age at the time of primary dislocation to be the most important prognostic factor in determining the risk of recurrence (Rowe, 1956; Henry & Genung, 1982; Hovelius et al., 1983a, b; Hovelius et al., 1996; Kralinger et al., 2002). Fewer studies concern the prognosis of function and morbidity after shoulder dislocation in elderly patients (Stayner et al., 2000). Our study showed the importance of including the elderly in future studies.

Hovelius et al. (1982) stated that nearly 50% of the people with primary dislocation never visited hospitals or were treated by a physician (Hovelius, 1982). In another study by Hovelius et al. concerning the Bristow–Latarjet procedure for recurrent anterior dislocation shoulder, it was reported that 37% of the 112 patients operated for dislocations had coped with their primary dislocation without consulting a physician or visiting a hospital (Hovelius et al., 1983a, b). The access to help for patients who need reduction of a dislocated shoulder is probably better in Norway today than in Sweden 30 years ago. However, an unknown number of patients do not seek medical attention and the true incidence for the general population remains unknown.

## Perspectives

The overall incidence of shoulder dislocations in Oslo was much higher than previously reported incidences of dislocations for the general population. The incidence of primary dislocations was also higher than in any earlier reported study for the general population but it was close to the reported incidence in Malmø, Sweden. A good knowledge of the demographic groups at risk can be useful when planning preventive strategies.
